# Clinical value of procalcitonin-to-albumin ratio for identifying sepsis in neonates with pneumonia

**DOI:** 10.1080/07853890.2023.2185673

**Published:** 2023-03-13

**Authors:** Tiewei Li, Xiaojuan Li, Zhiwei Zhu, Xinrui Liu, Geng Dong, Zhe Xu, Min Zhang, Ying Zhou, Jianwei Yang, Junmei Yang, Panpan Fang, Xiaoliang Qiao

**Affiliations:** a Zhengzhou Key Laboratory of Children’s Infection and Immunity, Children’s Hospital Affiliated to Zhengzhou University, Henan Children’s Hospital, Zhengzhou Children’s Hospital, Zhengzhou, P.R. China; b The Center of Henan Children’s Neurodevelopmental Engineering Research, Children’s Hospital Affiliated to Zhengzhou University, Henan Children’s Hospital, Zhengzhou Children’s Hospital, Zhengzhou, P.R. China; c Center of Laboratory Medicine, Women & Infants Hospital of Zhengzhou, Zhengzhou, P.R. China

**Keywords:** Procalcitonin-to-albumin ratio, sepsis, pneumonia, neonates

## Abstract

**Background:**

It is possible that neonates with pneumonia also have unrecognized sepsis. Identifying sepsis in neonates with pneumonia may cause some trouble for clinicians. This study aimed to evaluate the clinical value of the procalcitonin-to-albumin ratio (PAR) in identifying sepsis in neonates with pneumonia.

**Methods:**

We retrospectively included 912 neonates with pneumonia from January 2016 to July 2021. Clinical and laboratory data were collected from electronic medical records. Among neonates with pneumonia, 561 neonates were diagnosed with sepsis, according to the International Pediatric Sepsis Consensus. Neonates were divided into a sepsis group and a pneumonia group. A multivariate logistic regression analysis was used to evaluate whether PAR was a potential independent indicator for identifying sepsis in neonates with pneumonia. Receiver operating characteristic (ROC) curve analysis was performed to evaluate the diagnostic value of PAR in sepsis.

**Results:**

Neonates with sepsis have a higher PAR (*p* < 0.001). Correlation analysis showed that PAR was positively correlated with the level of C-reactive protein (*r* = 0.446, *p* < 0.001). Multiple logistic regression analysis showed that PAR was an independent predictor of the presence of sepsis in neonates with pneumonia. ROC curve analysis revealed that PAR had good power in identifying sepsis in neonates with pneumonia (area under curve (AUC) = 0.72, 95% confidence interval (CI), 0.68-0.75, *p* < 0.001).

**Conclusion:**

PAR can be used as a new biomarker to identify sepsis in neonates with pneumonia.

## Key Messages

- Compared with neonates with pneumonia, neonates with both pneumonia and sepsis had a higher PAR.
- PAR was a useful biomarker in distinguishing septic neonates from neonates with pneumonia.

## Introduction

The neonate’s lung is susceptible to microorganisms infection, which can lead to pneumonia [1,2]. Neonatal pneumonia is infectious lung disease with high morbidity and mortality worldwide [3,4]. The onset of neonatal pneumonia may be within hours of birth and part of a generalized sepsis syndrome. Neonatal sepsis is a severe bloodstream infection associated with a systemic inflammatory response and life-threatening organ system dysfunction [5]. Early recognition of sepsis and early treatment is encouraged by the Surviving Sepsis Campaign Physician’s management guidelines [6]. Recently, blood culture tests are the gold standard for diagnosing neonatal sepsis [7]. However, blood culture tests have a 48–72 h reporting delay and a low positive detection rate of pathogenic microorganisms [7]. Therefore, finding new biomarkers to distinguish sepsis from pneumonia in neonates is critical.

Infection/inflammation and malnutrition are common in patients with sepsis [8–12]. Procalcitonin (PCT) is a specific biomarker of infection/inflammation and can aid clinicians in diagnosing sepsis [13,14]. Albumin (ALB) is the most abundant circulating protein synthesized by the liver hepatocytes and is generally regarded as a nutritional indicator. Recent studies have demonstrated that ALB is closely correlated with inflammation, and hypoalbuminemia was frequently seen in patients with inflammatory diseases [15–18]. PCT to ALB ratio (PAR) is an index calculated as the PCT level divided by the ALB level, which can indicate both the body infection, inflammation, and nutritional status. Recently, Chen et al. [19] studied PCT and ALB and found that PAR was a predictive biomarker of mortality in adult patients with sepsis-induced acute kidney injury. However, so far, there is no research regarding the clinical value of PAR in identifying sepsis from pneumonia in neonates. This study aimed to investigate the clinical role of PAR in identifying these two clinical entities.

## Materials and methods

### Study population

This hospital-based retrospective study was conducted at Henan Children’s Hospital (Zhengzhou, China). A total of 912 neonates with pneumonia were included in this study from January 2016 to July 2021. The inclusion criteria for this study were (1) neonates aged ≤28 days and diagnosed with pneumonia and (2) neonates with complete clinical and laboratory data included in this study. This study excluded neonates with congenital liver defects, previous liver-related diseases, hematological system diseases, malignancies, major congenital malformations, or other inflammatory conditions. The study protocol complied with the Declaration of Helsinki and obtained the approval of the Hospital Ethics Review Board of Henan Children’s Hospital. All procedures performed in this study were undertaken as a part of routine clinical practice, and the data were anonymized. The requirement for informed consent was waived, considering the retrospective nature of the present study.

### Clinical definition

Neonatal pneumonia was mostly diagnosed based on clinical manifestations such as fever ( > 38.0 °C), respiratory distress, grunting, cough, hypothermia, hyperthermia, and chest X-ray suggestive of pneumonia. Neonatal sepsis was defined as a suspected or proven infection accompanied by ≥2 systemic inflammatory response syndrome (SIRS) criteria, one being an abnormal body temperature or leukocyte count according to the published International Pediatric Sepsis Consensus [20]. The SIRS criteria are as follows: (1) body temperature of more than 38.5 °C or less than 36 °C; (2) mean heart rate >2 SD above normal for age in the absence of external stimuli, or unexplained persistent elevation for children < 1 year old, or mean heart rate <10th percentile for age or unexplained persistent depression over a 0.5 hr period; (3) mean respiratory rate of more than 2 SD above normal for age or in the presence of mechanical ventilation; and (4) abnormal leukocyte count or >10% immature neutrophils. The diagnosis of pneumonia and sepsis was made by two study investigators.

### Data collection

The demographic and laboratory data at admission were collected from electronic medical records, including age, gender, body weight, body temperature, respiratory rate, heart rate, systolic blood pressure, diastolic blood pressure, and the levels of PCT, C-reactive protein (CRP), alanine aminotransferase (ALT), aspartate aminotransferase (AST), and ALB. Serum PCT concentrations were measured using the Cobas 8000 modular analyzer (Roche Diagnostic, Rotkreuz, Switzerland). CRP was quantified using the UPPER analyzer (Ultrasensitive CRP kit, Upper Bio-Tech, Shanghai, China). Levels of ALT, AST, and ALB were measured using the automatic Beckman biochemical analyzer (Beckman Coulter, California). Saturation of oxygen (SaO_2_) was measured using ABL800 FLEX analyzer (ABL800 FLEX, Bronshoj, Denmark). The fraction of inspired oxygen (FiO_2_) was estimated from the interface used for oxygen delivery.

In this study, CRP levels <0.8 mg/L were defined as a value of 0.7 mg/L. PCT level >100 ng/mL or <0.02 ng/mL were defined as 101 ng/mL and 0.01 ng/mL, respectively.

### Statistical analysis

Continuous variables are presented as mean ± SD for normally distributed variables, or medians (interquartile range) for non-normally distributed variables and are analyzed using independent *t*-tests or the Mann–Whitney *U* test. Categorical variables are expressed as a percentage and analysed using Chi-square tests. Spearman’s correlation method was used to evaluate the correlations between PAR and other clinical and laboratory indices. Binary logistic regression analysis was used to identify the independent risk factor for the presence of neonatal sepsis using the enter method. Variables with a P-value <0.05 in the univariate logistic analysis were included in the multiple logistic regression analysis. Receiver operating characteristic (ROC) curve analysis was performed to evaluate the predictive value of PAR for the presence of neonatal sepsis. Youden’s index was calculated (sensitivity + specificity − 1) to determine the optimal cut-off point. The area under the ROC curve (AUC) of the two variables was compared using Delong’s test. All data statistical analyses were performed using IBM SPSS version 24.0 (SPSS Inc., Chicago, Illinois, USA). A two-sided P value of less than 0.05 was considered statistically significant.

## Results

### Study population characteristics

This study included 511 neonates diagnosed with sepsis (Sepsis group) and 351 neonates without sepsis (Pneumonia group). Characteristics of the study population are shown in Table 1. Compared with neonates in the pneumonia group, neonates in the sepsis group were older, had a higher body temperature, respiratory rate, and heart rate, and had higher levels of PCT, CRP, ALT, and neutrophil count, and lower levels of ALB and SaO_2_/FiO_2_. Further analysis showed that neonates in the sepsis group had a low PAR and higher in-hospital mortality.

**Table 1.** Basic characteristics of study subjects by groups.

Variables	Pneumonia group (*n* = 351)	Sepsis group (*n* = 561)	*p*
Age (days)	7.0 (4.0, 14.0)	11.0 (5.0, 17.0)	<0.001
Aged 1–3 days, *n* (%)	63 (17.9%)	91 (16.2%)	0.498
Male, *n* (%)	204 (58.1%)	340 (60.6%)	0.456
Weight (kg)	3.3 ± 0.5	3.2 ± 0.7	0.003
Temperature (°C)	37.0 ± 0.5	37.4 ± 0.8	<0.001
Respiratory (rate/minute)	47.1 ± 8.5	50.7 ± 11.9	<0.001
Heart rate (bpm)	142.8 ± 17.4	151.9 ± 18.9	<0.001
SBP (mm Hg)	76.7 ± 7.9	76.0 ± 9.0	0.164
DBP (mm Hg)	46.7 ± 7.3	46.2 ± 8.6	0.381
Early onset sepsis	—	91 (16.2%)	—
Late-onset sepsis	—	470 (83.8%)	—
Biochemical parameters			
WBC (×10^9^ cells/L)	9.77 (7.94, 12.31)	10.09 (7.36, 14.91)	0.143
Neutrophil (×10^9^ cells/L)	4.1 (3.11, 6.05)	5.47 (3.11, 9.10)	<0.001
PCT (ng/ml)	0.15 (0.10, 0.28)	0.34 (0.14, 1.75)	<0.001
CRP (mg/L)	0.7 (0.7, 0.7)	0.7 (0.7, 17.8)	<0.001
ALT (U/L)	25.6 (20.0, 33.4)	28.8 (22.2, 38.7)	<0.001
AST (U/L)	37.8 (30.6, 51.7)	37.9 (27.9, 54.7)	0.554
ALB (g/L)	33.37 ± 4.14	30.08 ± 4.95	<0.001
SaO_2_/FiO_2_	4.63 (4.48, 4.67)	4.51 (4.16, 4.65)	<0.001
PAR (×10^-4^)	0.044 (0.029, 0.087)	0.113 (0.047, 0.649)	<0.001
In-hospital mortality, *n* (%)	0 (0)	12 (2.1%)	<0.001

Abbreviations: SBP: systolic blood pressure; DBP: diastolic blood pressure; PCT: procalcitonin; CRP: C-reactive protein; ALT: alanine aminotransferase; AST: aspartate aminotransferase; ALB: albumin; SaO2: Saturation of oxygen; FiO_2_: Fraction of inspired oxygen; PAR: procalcitonin to albumin ratio.

### Correlation between PAR and clinical parameters

As shown in Table 2, correlation analysis showed that PAR was negatively correlated with age and body weight and positively correlated with body temperature, respiratory rate, heart rate, CRP, ALT, and AST levels.

**Table 2.** Correlations between PAR and clinical parameters.

Variables	*r*	*P*
Age (day)	–0.224	<0.001
Weight (kg)	–0.146	<0.001
Temperature (°C)	0.132	<0.001
Respiratory (rate/minute)	0.160	<0.001
Heart rate (bpm)	0.126	<0.001
CRP (mg/L)	0.446	<0.001
ALT (U/L)	0.131	<0.001
AST (U/L)	0.157	<0.001

Abbreviations: PAR: procalcitonin to albumin ratio; CRP: C-reactive protein; ALT: alanine aminotransferase; AST: aspartate aminotransferase.

### Independence of PAR in identifying the presence of neonatal sepsis

Variables in univariate logistic analysis with a P value <0.05 were included in multivariable logistic regression analysis, which included age, body temperature, heart rate, respiratory rate, body weight, CRP, AST, and ALT levels. After adjusting these variables, PAR remained an independent indicator of sepsis in neonates with pneumonia (OR = 1.213, 95% CI 1.043-1.411, *p* = 0.012). Meanwhile, our data revealed that body temperature (OR = 2.216, 95% CI 1.628 − 3.015, *p* < 0.001), body weight (OR = 0.518, 95% CI 0.397 − 0.677, *p* < 0.001) and CRP (OR = 1.072, 95% CI 1.046 − 1.099, *p* < 0.001) were independent indicators for the presence of sepsis (Table 3).

**Table 3.** Regression analyses to determine the independent predictor of neonatal sepsis.

Variables	Univariate		Multivariate*	
	OR (95% CI)	*P*	OR (95% CI)	*P*
Age (day)	1.048 (1.028 − 1.068)	<0.001	1.043 (1.017 − 1.068)	0.001
Temperature (°C)	2.357 (1.869 − 2.974)	<0.001	2.216 (1.628 − 3.015)	<0.001
Heart rate (bpm)	1.025 (1.017 − 1.033)	<0.001	1.004 (0.994 − 1.015)	0.435
Respiratory rate (rate/minute)	1.036 (1.022 − 1.051)	<0.001	1.009 (0.991 − 1.026)	0.333
Weight (kg)	0.738 (0.596 − 0.915)	0.006	0.518 (0.397 − 0.677)	<0.001
CRP (mg/L)	1.083 (1.057 − 1.109)	<0.001	1.072 (1.046 − 1.099)	<0.001
AST (U/L)	1.003 (1.000 − 1.006)	0.034	1.003 (0.996 − 1.010)	0.462
ALT (U/L)	1.011 (1.004 − 1.017)	0.001	1.002 (0.999 − 1.005)	0.263
PAR (×10^–4^)	1.512 (1.247 − 1.833)	<0.001	1.213 (1.043 − 1.411)	0.012

*Adjusted for age, temperature, heart rate, respiratory rate, weight, CRP, AST, and ALT.

Abbreviations. PAR: procalcitonin to albumin ratio; CRP: C-reactive protein; ALT: alanine aminotransferase; AST: aspartate aminotransferase.

### Diagnostic value of PAR in neonatal sepsis

**Figure 1.**
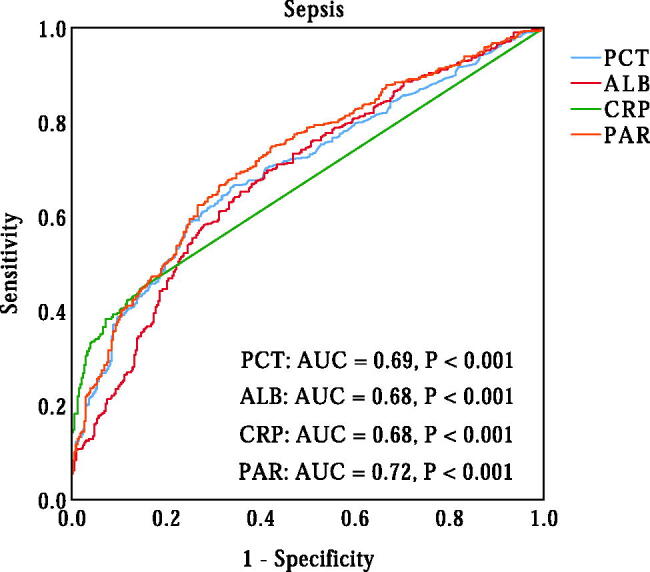
ROC curve of PCT, ALB, CRP, and PAR in identifying neonatal sepsis. PCT: procalcitonin; ALB: albumin; CRP: C-reactive protein; PAR: procalcitonin-to-albumin ratio.

**Figure 2.**
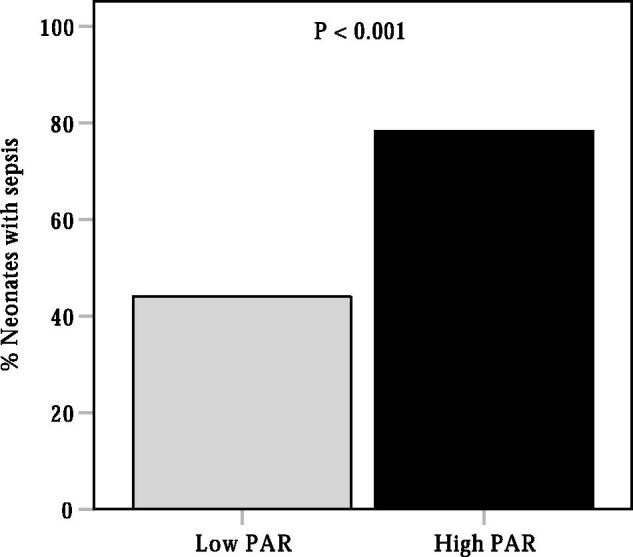
The distribution of neonatal sepsis into high or low PAR groups. PAR, procalcitonin-to-albumin ratio.

A ROC curve analysis was performed to evaluate the ability of PAR in identifying the presence of neonatal sepsis. As shown in Figure 1, the AUC for PAR in predicting sepsis was 0.72 (95% CI, 0.69–0.76, *p* < 0.001), which was significantly higher than the AUC for PCT (AUC = 0.69, 95% CI, 0.66–0.73, *p* < 0.001) (*p* < 0.05). The optimal cut-off value of PAR in identifying the presence of neonatal sepsis was 0.070 × 10^−4^, with 64% sensitivity and 73% specificity. Additionally, we also evaluated the ability of PCT, ALB and CRP to predict neonatal sepsis. The AUCs for PCT, ALB and CRP were 0.69 (95% CI, 0.66–0.73, *p* < 0.001), 0.68 (95% CI, 0.65–0.72, *p* < 0.001) and 0.68 (95% CI, 0.64–0.71, *p* < 0.001), respectively, which were lower than the AUC for PAR in predicting sepsis in neonates with pneumonia (*p* < 0.05). According to the cut-off value of PAR, neonates were divided into two groups: the low PAR group (PAR ≤ 0.070) and the high PAR group (PAR > 0.07). As shown in Figure 2, the prevalence of neonatal sepsis was significantly higher in the high PAR group than that in the low PAR group (78.2% vs. 45.0%, *p* < 0.001).

## Discussion

Sepsis is a systemic inflammatory response syndrome caused by infection and accompanied by pathological inflammation and organ system dysfunction, which seriously threatens human health, especially in newborns [21]. Compared with adults, neonates were more susceptible to being infected by pathogenic microorganisms and had a high risk of developing pneumonia and sepsis owing to their immature immune systems [22]. Neonatal pneumonia is a relatively common lung infection. Its onset may be within hours of birth and may progress to neonatal sepsis [1]. Neonates with pneumonia may also have unrecognized sepsis. If neonates with both pneumonia and sepsis are not treated for sepsis, they will not benefit from the early treatment of sepsis [6]. Therefore, distinguishing septic neonates from neonates with pneumonia is critical for the effective therapeutic management of sepsis in neonates.

PCT is a 116-amino acid peptide produced by the thyroid C-cells and maintained at a low circulating concentration (<0.05 ng/ml) in healthy individuals [23]. PCT levels can increase significantly when there is a bacterial infection or some form of tissue injury. Studies have demonstrated that PCT is a very useful biomarker for diagnosing sepsis and positively correlates with the severity of sepsis in adults and children (including neonates) [24–28]. ALB is the most abundant protein found in the blood produced by the liver and is commonly used as a clinical index of nutrition to evaluate the nutritional status of the body [29]. However, some studies reported that ALB was closely associated with inflammation and hypoalbuminemia was frequently observed in patients with inflammatory diseases [15,16,29,30]. Yang et al. [31] reported that hypoalbuminemia was common among neonates with sepsis and neonates with low ALB levels had a poorer prognosis. In this study, our data showed that neonates with sepsis had a higher PCT level and a lower ALB level, indicating that PCT and ALB may aid in the earlier identification of sepsis in neonates with pneumonia.

PAR is an index based on serum PCT and ALB levels that are calculated as the PCT level divided by the ALB level and can indicate both the body infection/inflammation and nutritional status. Studies reported that ALB to PCT ratio could be used as a sensitive early marker of nosocomial bloodstream infection in patients with intracerebral hemorrhage and a higher PAR level is strongly correlated with higher mortality in patients with sepsis-induced acute kidney injury [19,32]. Luo et al. [33] revealed that PAR is also an early diagnostic predictor in discriminating urosepsis from patients with febrile urinary tract infections. However, whether PAR can distinguish sepsis from pneumonia in neonates remains unknown.

In the present study, we first evaluate the clinical value of PAR in identifying septic neonates from neonates with pneumonia. Our data showed that neonates with both pneumonia and sepsis had a higher level of PAR. Further analysis revealed that PAR showed a positive correlation with body temperature, respiratory rate, heart rate, and the level of CRP, ALT, and AST. Multivariate analysis revealed that PAR was an independent biomarker in identifying septic neonates from neonates with pneumonia.

Our study also has several limitations. First, neonatal sepsis was diagnosed based on clinical features rather than a positive blood culture test result. Therefore, the true incidence rate of neonatal sepsis may be underestimated or overestimated. Second, because this is a retrospective single-center study, the findings should be replicated by multicenter clinical studies. Third, we did not further divide them into early-onset sepsis and late-onset sepsis, because the number of neonates aged 1–3 days was relatively small. Finally, we only measured PAR at admission and serial PAR measurements may be more useful in evaluating the clinical value of PAR for identifying sepsis in neonates with pneumonia.

## Conclusions

In conclusion, our study demonstrated that PAR was a useful biomarker in identifying septic neonates from neonates with pneumonia.

## Data Availability

The data used to support the findings of this study are available from the corresponding author upon request.
